# Swimming coaches' perceptions and practices on periodization, performance monitoring, and training management

**DOI:** 10.3389/fspor.2025.1642020

**Published:** 2025-08-22

**Authors:** Cristina Cano-Cuartero, Alejandro López-Hernández, Sergio Rodríguez-Barbero, José María González-Ravé

**Affiliations:** ^1^ Sport Training Laboratory, University of Castilla-La Mancha, Toledo, Spain; ^2^ Universidad Internacional de La Rioja, Facultad Ciencias de la Salud y Escuela de Doctorado, Logroño, Spain

**Keywords:** swimming, coaching, periodization, training load, tapering, performance monitoring

## Abstract

**Introduction:**

This study examined the beliefs and practices of Spanish national swimming coaches regarding season planning, aiming to gain a deeper understanding of how they organize training throughout the year.

**Methods:**

A total of 18 coaches participated and were classified based on the performance level of their swimmers: World Class (27.8%), Elite (11.1%), and National (72.2%). A validated questionnaire was used to gather information on training structure, session management, and feedback strategies.

**Results:**

The most reported planning model was traditional periodization (35.3%), typically structured into general, specific, and competitive phases, with individualized tapering strategies lasting between 7 and 21 days. While most coaches (89.9%) did not involve swimmers in the planning process, they did consider contextual factors such as academic and personal schedules (94.4%). Coaches emphasized strength-endurance and coordination work during early phases, shifting toward speed-strength and sprint capacity in the competitive phase. Training sessions were commonly adjusted based on objective and subjective indicators (83.3%), including heart rate (77.8%) and perceived exertion (55.6%). Feedback was mostly provided during training and addressed psychological (72.2%) and technical aspects (38.9%). Recovery strategies included active rest (22.7%), professional guidance (22.7%), and collaborative planning between coach and swimmer (61.1%). Performance assessments were conducted using tools such as the force-velocity profile (44.4%), one-repetition maximum test (22.2%), countermovement jump (16.7%), and swim-specific sets (7 × 200 m) (22.2%), although one-third of coaches did not use formal testing. While the limited sample size restricts the generalizability of findings, the results offer valuable insight into how experienced coaches conceptualize and manage the training process.

**Discussion:**

These findings highlight the importance of individualized planning, continuous monitoring, and athlete-context integration in high-performance swimming coaching.

## 1 Introduction

Season planning in swimming is crucial for performance, as it organizes workload, structures training, and adapts methods to each athlete's needs (1, 2). Coaches play a critical role in organizing and executing this planning. Their knowledge, experience, and perceptions about effective strategies directly influence how training programs are designed and implemented (3).

One of the fundamental principles guiding training organizations is periodization, which refers to the systematic structuring of training into macrocycles, mesocycles, and microcycles, to optimize physical, technical, and psychological adaptations (4, 5). In swimming, coaches often rely on traditional periodization models (6), although their application is frequently adjusted based on contextual factors, coaching experience, and athlete needs (7). Effective in-season planning requires balancing training volume and intensity to promote peak performance (8). Understanding how coaches interpret and apply periodization is therefore essential to bridging the gap between theoretical frameworks and real-world practices.

Recent studies have begun to explore this gap. For instance, Dalamitros et al. (9) analyzed planning and monitoring strategies among a large and internationally diverse sample of swimming coaches. Their findings revealed considerable variability in the implementation of periodization and evaluation tools, shaped by factors such as training environment, resource availability, and competition level. While these large-scale studies provide important global insights, there is still a need for more in-depth, context-specific research to understand how planning decisions unfold within national systems.

In parallel, several authors have linked periodization not only to structural aspects of training but also to broader constructs such as training quality. According to Sandbakk et al. (10), training quality depends on how and why the season is planned, organized, and developed to achieve a performance goal. Some authors define training quality as the athlete's ability to complete a session at the optimal level (10, 11). This concept can be analyzed from different angles: the quality of the holistic process (goal-setting, environmental adaptation, and method selection), and the quality of the individual session (pre-session planning, load management, feedback, and recovery). Since periodization provides the framework that shapes these dimensions over time, coaches' views on periodization are closely intertwined with how they understand and pursue training quality. Their choices reflect philosophical and practical beliefs about effective preparation (3, 10).

Coaches also face multiple factors that influence how they plan the season. Ritchie and Allen (12) observed that during major competitions, such as the Olympic and Paralympic Games, coaches often adopt a more observational and adaptive role, granting athletes greater autonomy while making tactical adjustments. Similarly, Hellard et al. (13) demonstrated that increasing the total training load, particularly through endurance and strength training, in the weeks leading up to the competition can enhance performance in sprint events.

Additional variables, such as swimmer age, developmental stage, competitive demands, and institutional constraints (e.g., clubs or federations), also influence planning decisions (14). Furthermore, discrepancies between the coach's intended training load and the swimmers' perceived effort may affect performance outcomes (15). As Johns et al. (7) suggest, uncovering the often-invisible and relational aspects of coaching requires research approaches sensitive to context and nuance. In fact, previous studies have emphasized how positivist or purely quantitative methods often fail to capture the dynamic and situated nature of coaching practice (16).

Given that coaches' perceptions are shaped by personal experience, specific constraints, and social interactions, adopting an interpretive approach enables researchers to explore the meaning that coaches assign to key concepts such as periodization (7). This aligns with theoretical perspectives that define coaching as a relational and adaptive process, where beliefs and context jointly shape planning decisions (17).

Despite growing evidence supporting the benefits of structured periodization models (13, 18), and recent systematic reviews that describe their application across both conventional and para-swimming contexts (19), there remains a lack of research examining how swimming coaches perceive and apply these models in daily practice. Moreover, coaches' beliefs about periodization are closely related to broader notions of training quality, athlete development, and contextual adaptation (7, 10). Given the relational and adaptive nature of coaching (17), understanding how coaches conceptualize and implement periodization is essential to bridging the gap between theoretical models and real-world practice.

Therefore, this study aimed to explore swimming coaches' perceptions and beliefs about season planning, with a particular focus on how they define and implement periodization strategies within their coaching practice.

## 2 Materials and methods

### 2.1 Design

This study employed a non-experimental, cross-sectional, and descriptive design. “*Non-experimental*” means that no variables were manipulated, and the researchers did not assign participants to experimental or control groups. “*Cross-sectional*” refers to the fact that data were collected at a single point in time, rather than longitudinally across multiple periods. “*Descriptive*” indicates that the primary aim was to observe and describe the perceptions and practices of swimming coaches without testing specific causal hypotheses.

A structured questionnaire was used to explore coaches' perceptions, beliefs, and methods related to season planning, training load management, feedback strategies, and performance monitoring. The questionnaire was adapted for swimming and reviewed by three swimming coaching professionals with more than ten years of experience, as well as two researchers with substantial scientific production and citation metrics in coaching and sports periodization. This questionnaire collected data on coaches' perceptions, beliefs, and training practices for seasonal planning. This design allows for analysing the relationships between variables at a single point in time without manipulating environmental conditions. The questionnaire is based on previous studies (1, 3).

### 2.2 Sample

The sample consisted of 18 national swimming coaches classified as follows:

• Athlete performance level: World Class (*n* = 5, 27.8%), Elite (*n* = 2, 11.1%), National level (*n* = 13, 72.2%).
• Years of coaching experience: less than 10 years (*n* = 5, 27.8%), more than 10 years (*n* = 13, 72.2%).
• Academic qualifications: Certified Swimming Coach (*n* = 14, 77.8%), Degree in Sports Sciences (*n* = 5, 27.8%), Master's in Sports Sciences (*n* = 6, 33.3%), PhD in Sports Sciences (*n* = 1, 5.6%).
• Age groups coached: Age group (9–12 years), Youth (13–15 years), Junior (16–18 years), Senior (>19 years).

The sample was selected intentionally, with the only requirement being to include coaches with relevant and representative experience in national and international competitions. Coach characteristics are presented in Table 1.

**Table 1:** Coach characteristics.

Characteristic	*N* (%)
Country	
Spain	18 (100%)
Years of experience	
Less than 10 years	5 (27.8%)
More than 10 years	13 (72.2%)
Education level	
Certified Swimming Coach	14 (77.8%)
Degree in Sport Sciences	5 (27.8%)
Degree in Education	2 (11.1%)
Degree in Other Fields	1 (5.6%)
Master in Sport Sciences	6 (33.3%)
PhD in Sport Sciences	1 (5.6%)
Senior Swimming Coach	1 (5.6%)
PhD Candidate	1 (5.6%)
Bachelor in Sport Sciences	1 (5.6%)
Age Group Coached	
Age group (9–12 years)	5 (27.8%)
Youth (13–15 years)	6 (33.3%)
Junior (16–18 years)	14 (77.8%)
Senior (19+ years)	14 (77.8%)
Athlete Performance Level^a^	
World Class (Level 5)	5 (27.8%)
Elite (Level 4)	2 (11.1%)
National (Level 3)	13 (72.2%)
Disciplines Coached	
Freestyle (50–1,500 m)	7 (38.9%)
Backstroke (50–200 m)	3 (17.6%)
Breaststroke (50–200 m)	4 (22.2%)
Butterfly (50–200 m)	2 (11.1%)
Individual Medley (200–400 m)	2 (11.1%)
Freestyle Relays	0
Medley Relays	0

^a^ Athlete performance level classification (20).

Some participants provided more than one response to these questions; therefore, the total number of responses exceeded the number of participants (*n* = 18). Percentages are calculated based on the total number of responses.

This study was approved by the Social Research Ethics Committee of the University of Castilla-La Mancha (CEIS-728331-D9H0).

### 2.3 Variables

The study seeks to answer the following research questions:

(a) What are swimming coaches' perceptions and beliefs about periodization and season planning?
(b) How do these beliefs influence their methodological practices, including training session management, tapering, and performance monitoring?
  Based on these questions, the main variables examined were:
  • Coach background: Demographic data, experience, qualifications, and competitive level of swimmers coached.
  • Holistic training process: Coaches' definitions of training quality, periodization models used, phase structure, tapering strategies, and objectives and resources for strength and swimming training across different phases.
  • Training session quality: Session planning and adjustment, monitoring methods (objective and subjective), feedback strategies, recovery protocols, and performance testing.

### 2.4 Materials and instrumentation

The primary data collection tool was a structured questionnaire divided into three sections:

1. Personal Information: Collects sociodemographic data and the professional background of the coach.
2. Holistic Process Quality: Focused on the quality of the planning process, including periodization and objectives for each training phase.
3. Session-Level Quality: Aimed at collecting information on session management, load monitoring, and feedback mechanisms.

The data were collected online via a custom-designed “ad hoc” questionnaire. The original questionnaire developed by Agudo-Ortega, Salinero et al. (1) was adapted for this study. Specifically, items were revised to better fit the Spanish competitive swimming context. The adapted version was reviewed by a panel of three experienced swimming coaches (with more than 10 years of experience) and two researchers with expertise in coaching and periodization (3). Based on their feedback, a total of 9 items were modified to improve clarity, contextual relevance, and alignment with the terminology commonly used in swimming coaching in Spain. No items were added or removed. The modified questions included “*Item 7 in* Section 1*; Items 11, 15, and 18 to 26 in* Section 2*; and Items 7 and 18 in* Section 3”. The final version was considered to have satisfactory content validity for the target population.

The questionnaire was distributed through the Google Forms platform. Alongside the invitation to participate and the link to the questionnaire, coaches received detailed information about the study and instructions for completing the questionnaire concerning a full year of training for their most prominent athlete. The questionnaire is available as Supplementary material S1. Before accessing the questionnaire, participants were presented with a detailed information sheet outlining the study's objectives, the voluntary and anonymous nature of participation, and their right to withdraw at any time. The form also included explicit statements regarding compliance with the General Data Protection Regulation (GDPR) (EU 2016/679) and national data protection laws. Only participants who gave informed consent by continuing beyond this screen were able to complete the questionnaire.

### 2.5 Data analysis

Questionnaire data were exported to an Excel spreadsheet and analyzed using a mixed-methods approach. Closed-ended responses were treated quantitatively, and we calculated absolute and relative frequencies (*n* and %) as well as means and standard deviations (mean ± SD) for Likert-type scales and categorical responses. Open-ended questions were designed to explore coaches' interpretations and practical approaches to various elements of season planning and training. These included topics such as definitions of training quality, structure of the season, tapering protocols, workload monitoring, warm-up routines, and feedback strategies. Example questions included: *“What do you understand by training quality?” and “Can you describe your tapering reduction protocol?”*. For these questions, we conducted a conventional qualitative content analysis following the approach outlined by Hsieh and Shannon (21). Two researchers independently reviewed the raw responses, generated initial codes inductively, and grouped similar codes into broader themes. Discrepancies were resolved through consensus to ensure reliability. The goal of this analysis was to uncover shared beliefs, practices, and contextual challenges as expressed in coaches' own words. Themes were presented descriptively in the results, supported by anonymized illustrative quotes. Figure design was performed using GraphPad Prism v9.3.1. (GraphPad Software, San Diego, CA, USA).

## 3 Results

### 3.1 Characteristics of the planning process

Most coaches (88.9%, *n* = 16) plan the season individually, without input from their swimmers. Only 2 coaches (11.1%) engage in co-design planning with their athletes. Nearly all coaches (94.4%, *n* = 17) consider the swimmer's environment, such as work schedules, family commitments, academic responsibilities, and free time, when planning the season.

Coaches reported using various periodization models (Table 2). The most mentioned was traditional periodization, reported by 6 coaches (35.3%). Initially, 5 coaches used block periodization, and 1 coach specifically mentioned the ATR model. As the ATR model is considered a specific form of block periodization, we grouped these responses under the same category. Thus, a total of 6 coaches (35.3%) reported using block periodization approaches, making their prevalence equal to that of traditional periodization in our sample. The following most used models are reverse block loading periodization (5.6%, *n* = 1). Finally, two coaches (11.2%) reported using a hybrid periodization model, defined as a flexible combination of elements from both traditional and block approaches. These models allow adaptation throughout the season based on competition schedules, swimmer readiness, or facility availability. For example, coaches might start the season with traditional linear progression and then shift to a block structure during competition phases. Seasonal training follows a common structure divided into phases. Coaches reported:

**Table 2:** Types of periodization models used by coaches according to their level.

Coach Level	Periodization models				
	Traditional	Block	Reverse	Hybrid	Total
World Class	2	1	1	1	5
Elite	0	0	0	2	2
National	5	6	1	1	13
Total	**7**	**7**	**2**	**4**	**20**

“Introductory phase lasting 2–3 weeks, general phase lasting 3–4 weeks, specific phase lasting 2 weeks, and competitive phase lasting approximately 6 weeks” (Coach 5, national level)

“Preseason (introductory) lasting 4 weeks. The general phase lasts 8 weeks. Specific- Competitive lasting 4 weeks. Transition phase lasting 2 weeks” (Coach 7, world-level).

Tapering durations most used by coaches were 7–14 days (27.8%, *n* = 5), or 17–21 days (27.8%, *n* = 5), followed by 0–7 days (22.2%, *n* = 4) and 21–28 days (22.2%, *n* = 4).

Coaches agreed that tapering involves gradually reducing training volume while maintaining or increasing intensity. Typically beginning 10–21 days before competition, tapering volume reductions range from 30% to 60%, tailored to the athlete's form and competition importance. Intensity is maintained or increased, focusing on race-specific technique and pacing. Across all levels, tapering is adapted to each swimmer to maximize peak performance.

**Figure 1:**
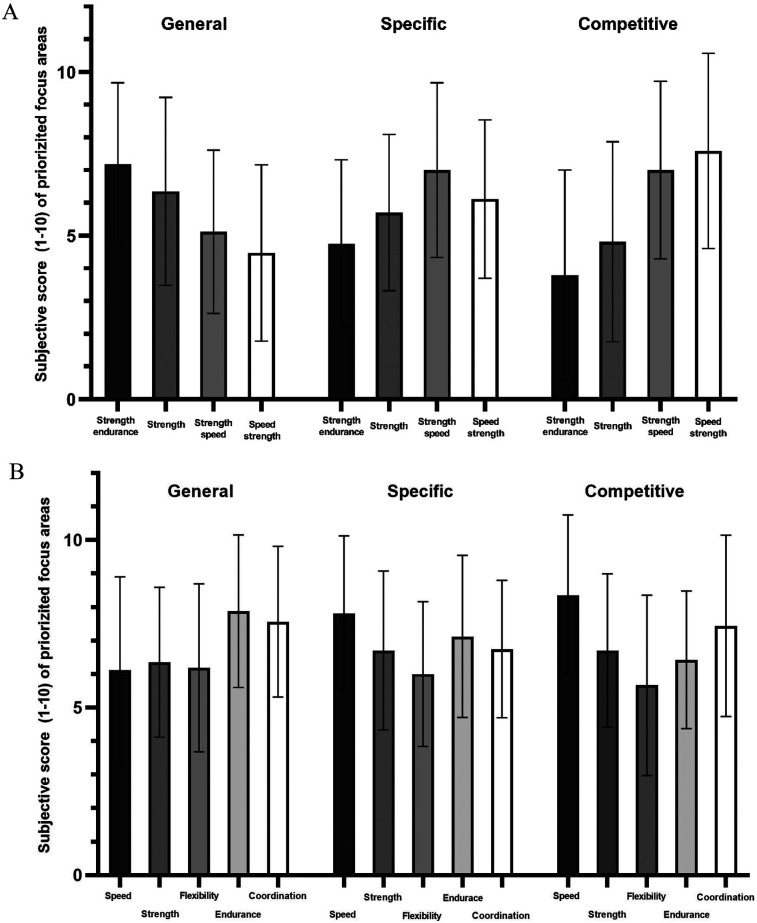
Development of strength **(A)** and swimming **(B)** capacities during the different phases of a macrocycle of national swimming coaches.

Figure 1 illustrates the focus on strength (A) and swimming capacities (B) across macrocycle phases. These values represent coaches' ratings of the importance of each training focus area, measured on a “*Likert scale*” from 0 (not important at all) to 10 (extremely important). During the general phase, emphasis is placed on strength-endurance (7.2 ± 2.5), general strength (6.4 ± 2.9), coordination (7.3 ± 2.4), endurance (7.6 ± 2.5), and speed (6.2 ± 2.3). In the specific phase, focus shifts to strength-speed (6.9 ± 2.7), speed-strength (6.1 ± 2.4), swim-specific speed (7.8 ± 2.3), and swim-specific endurance (7.1 ± 2.4). In the competitive phase, coaches emphasize speed-strength (7.5 ± 3.0), strength-speed (7 ± 2.7), speed (8.4 ± 2.4), and coordination (7.4 ± 2.7).

### 3.2 Practices in training session management

Most coaches conduct in-person monitoring of training sessions (88.3%, *n* = 16), while a smaller portion uses a hybrid approach (11.1%, *n* = 2), combining in-person and online elements. None of the coaches reported using a fully online approach.

**Warm-up protocols** generally follow a consistent structure. Coaches describe these protocols as:

“A 10–15-minute dry warm-up at the water's edge with mobility, culminating in muscle activation. In the water, sensations with technique progressions, culminating in speed activation” (Coach 5, world-level).

“1. Mobility. 2. Injury prevention. 3. Activation and core. 4. Easy swimming. 5. Progressive swimming and speed” (Coach 9, national level).

Then technical drills in the water, and finally progressive swim reps with speed activation.

**Feedback** is primarily delivered during sessions (72.2%, *n* = 13), followed by after sessions (22.2%, *n* = 4), and to a lesser extent, offers pre-session information (5.6%, *n* = 1).

**Figure 2:**
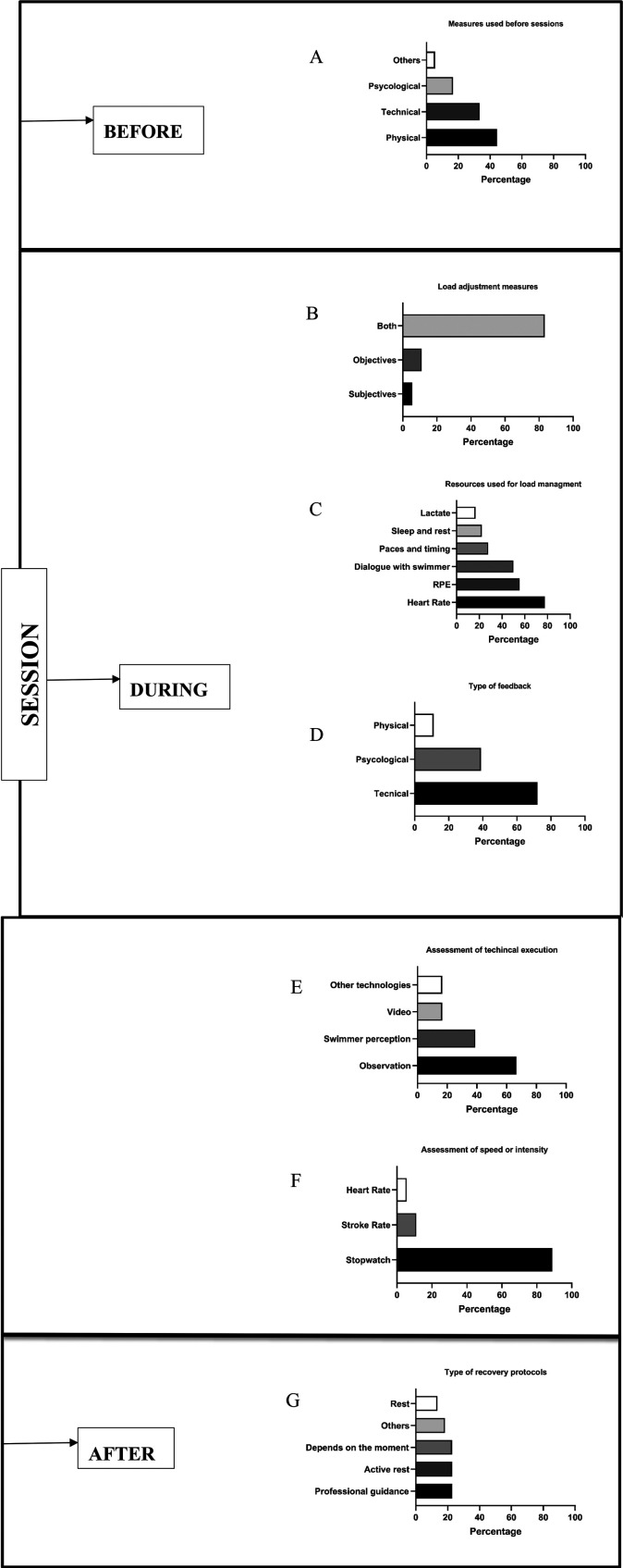
Management of national swimming coaches' sessions. Measures used before **(A)**, during **(B–F)**, and after sessions **(G)**.

**Adjustments before sessions** are common. 83.3% (*n* = 15) of coaches modify their planned sessions based on pre-session measurements. The remaining (16.7%, *n* = 3) do not collect pre-session data and therefore do not adjust their sessions. Figure 2A shows that adjustments are mainly based on physical (44.4%, *n* = 8) and technical (33.3%, *n* = 6) indicators, followed by psychological factors (16.6%, *n* = 3) and other variables (5.6%, *n* = 1).

**During sessions**, 83.3% (*n* = 15) of coaches use both objective and subjective measures to adjust training loads, while 11.1% (*n* = 2) rely only on objective data, and 5.6% (*n* = 1) on subjective feedback (Figure 2B). Figure 2C highlights the tools used for monitoring training load. Heart rate (77.8%, *n* = 16), rate of perceived exertion (55.6%, *n* = 12), athlete feedback (50%, *n* = 9), sleep/rest patterns (22.2%, *n* = 4), pacing and split times (27.8%, *n* = 5), and lactate testing (16.7%, *n* = 3). Figure 2D illustrates the focus areas of in-session feedback, categorized into psychological aspects (72.2%, *n* = 13), technical aspects (38.9%, *n* = 7), and physical aspects (11.1%, *n* = 2). Figure 2E outlines tools for evaluating technical execution: coach observation (66.7%, *n* = 12), athlete self-perception (38.9%, *n* = 7), video analysis (16.7%, *n* = 3), and other methods (16.7%, *n* = 3). Figure 2F presents how swim speed/intensity is measured: stopwatches (88.9%, *n* = 16), stroke rate (11.1%, *n* = 2), and heart rate (5.6%, *n* = 1).

**In the post-session**, 16 coaches (88.9%) assess the gap between planned and actual performance and adjust the next session accordingly. Only 11.1% (*n* = 2) don't adjust upcoming sessions.

**Recovery protocols** are mostly designed in collaboration between coach and athlete (61.1%, *n* = 11), while 27.8% (*n* = 5) are coach-designed only, and 11.1% (*n* = 2) report not using recovery protocols. Figure 2G shows the types of recovery methods used: professional guidance (22.7%, *n* = 5), active rest (22.7%, *n* = 5), total rest (13.6%, *n* = 3), situational approaches (18.2%, *n* = 4), and other strategies (22.7%, *n* = 5).

**Figure 3:**
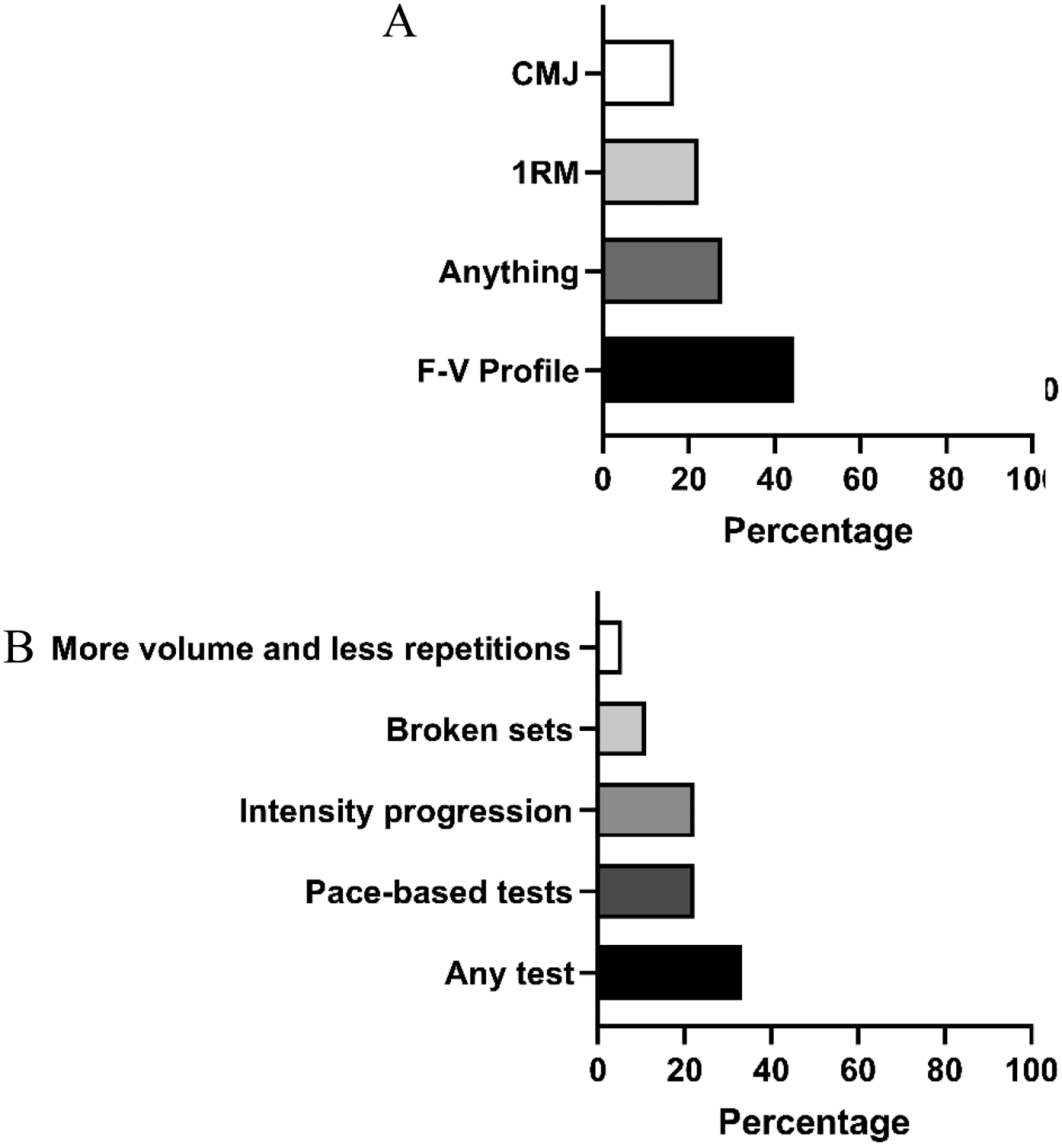
Assessment tools developed by national swimming coaches to monitor the progression of strength **(A)** and speed **(B)** performance.

To monitor strength and conditioning performance during testing days, coaches used various assessment tools: Figure 3A shows that the force-velocity profile measured with an encoder was the most used method (44.4%, *n* = 8), followed by 1RM (22.2%, *n* = 4), and countermovement jump (16.7%, *n* = 3). A notable percentage (27.8%, *n* = 5) did not use any tests. Figure 3B indicates that for swim performance evaluation, progressive intensity tests (7 × 200 m) and pace-based test sets (22.2% each, *n* = 4) were most common, followed by broken sets (11.1%, *n* = 2) and reduced-rep distance increases (5.6%, *n* = 1). Still, 33.3% (*n* = 6) reported not using any performance tests.

### 3.3 Qualitative findings from open-ended responses

A qualitative content analysis of the open-ended responses revealed several recurring themes that offer deeper insight into coaches' planning philosophies and contextual limitations:

**Autonomy *vs.* Structure**: This theme captures how coaches navigate the gap between planned periodization frameworks and the need to adapt based on athletes' readiness, health, and unforeseen changes.

“Planning is essential, but it's rarely followed exactly. The athlete's daily condition often overrides the initial structure.” (Coach 4, National level)

“I try to follow the periodized plan, but adjustments are constant—especially in the specific and taper phases. Illness, fatigue, or external factors often shift priorities.” (Coach 9, World level)

This suggests that while coaches value structured periodization, its application is often fluid and responsive, reinforcing the idea that planning is a dynamic rather than static process.

**Contextual Constraints**: Many coaches described how external factors—such as pool access, academic calendars, or club schedules—can limit the execution of ideal training plans.

“Our plan must revolve around school schedules and the availability of facilities. Ideal periodization is often compromised.” (Coach 5, National level)

“We have no control over some aspects—like when the pool is available or when meets are scheduled. This forces us to shift the structure constantly.” (Coach 2, Elite level)

These insights show how periodization choices are often shaped more by logistical realities than by theoretical preferences.

**Monitoring Overload and Simplicity**: This theme reflects a philosophical divide, with some coaches embracing detailed performance monitoring while others resist it, preferring intuitive, athlete-centered adjustments.

“I rely on heart rate and how the swimmer feels. Too many tools can distract from the athlete.” (Coach 1, World level)

“We track some data, but I value conversation and observation more. Numbers help, but they don’t tell the full story.” (Coach 6, National level).

**Athlete Exclusion from Planning**: Although most coaches designed training independently, some acknowledged a growing recognition of the value of athlete input.

“The swimmer's input is minimal. Perhaps this should change in the future.” (Coach 7, National level)

“I try to involve them more during tapering or when they express fatigue, but overall, I still lead the planning.” (Coach 3, National level)

This theme reveals a potential shift in coaching culture toward more collaborative planning, although traditional coach-led structures still dominate.

## 4 Discussion

This study aimed to explore how experienced swimming coaches conceptualize and apply season planning strategies, particularly about periodization, training quality, and contextual constraints. By combining closed- and open-ended questions in a structured questionnaire, this exploratory design enabled the capture of both standardized planning practices and the underlying reasoning behind them.

Building on the data collected, the results reveal a nuanced planning landscape in which coaches balance tradition with adaptability. Traditional and block periodization models were the most used, with each reported by 35.3% of participants. This dual preference suggests that both models are perceived as viable, depending on the context, swimmer profile, and training goals. Traditional periodization typically involves a structured progression across general, specific, and competitive phases, with a focus on preparing swimmers to reach peak condition for a main competition (6). This approach typically includes a general phase focused on building aerobic endurance and technical foundations (2). For instance, Ferreira et al. (18) reported a 14.6% improvement in 400 m freestyle performance after a year-long program using three macrocycles in young swimmers. In contrast, block periodization offers greater flexibility for targeting specific physiological or technical adaptations. Evidence on its effectiveness remains mixed. While some studies report improvements in body composition and stroke technique, others show no significant gains in performance (22). This may help explain why both models are still widely adopted in practice. This ambiguity may explain why coaches in our sample reported using block periodization selectively, especially when addressing short-term performance needs or swimmer specialization.

Building upon these findings, it is important to consider how cultural and structural factors may shape the planning models chosen by coaches. While Spanish coaches leaned heavily toward traditional approaches, this may reflect a broader adherence to rigid, coach-led planning frameworks driven by institutional norms. However, it is possible that pragmatic considerations—*such as alignment with club routines or familiarity from coach education pathways*—may play a role in shaping these preferences. This contrasts with international practices in countries such as Australia, the United States, and Norway, where more collaborative, evidence-based models are gaining ground (23, 24). These approaches allow for more targeted physiological adaptations and are often integrated with technology-supported monitoring tools and athlete feedback systems. For example, the Long-Term Athlete Development (LTAD) framework has become more influential in athlete-centered systems like those in Canada and Scandinavia, where swimmer input and co-construction of plans are more common (25). Moreover, only 11.1% of coaches in our study reported involving athletes in planning decisions—an indicator of limited athlete agency that may reflect hierarchical coaching traditions. This low level of collaboration contrasts with emerging literature that advocates for shared decision-making as a performance-enhancing practice (7). These contextual observations reinforce the importance of understanding coaching practice as a socially situated activity rather than a purely technical endeavor. This aligns with previous research suggesting that coaching beliefs are shaped by a combination of formal knowledge, experiential learning, and the social environment in which coaching occurs (17, 26, 27). These dynamics influence how coaches construct and apply training models in practice. In line with this, Nugent et al. (28) found that expert swimming coaches emphasize quality over quantity, relying on individualization and contextual adaptation to optimize performance outcomes.

In addition to choosing a periodization model, coaches in this study consistently structured their season across three key phases: general, specific, and competitive. In the general phase, emphasis was placed on developing aerobic capacity, technical foundations, strength-endurance, and coordination, which are essential for injury prevention and long-term performance development (6). The specific phase shifted the focus toward strength-speed, technical-tactical refinement, and race-specific endurance (3, 29). Finally, the competitive phase prioritized speed-strength, neuromuscular coordination, and technical precision (3, 30). Coaches reported including a tapering period of 2–4 weeks, during which training volume was reduced, and intensity and specificity increased, tailored to each swimmer's needs (6, 31). This systematic progression is aligned with current best practices in swimming and other individual sports (3, 13, 32).

In addition to macrostructural planning, coaches reported applying consistent monitoring and feedback practices across training cycles. Monitoring was conducted before, during, and after sessions using both objective (e.g., heart rate, time trials) and subjective (e.g., perceived effort, swimmer feedback) indicators. This dual approach to training load aligns with contemporary literature emphasizing the integration of internal and external load metrics (32, 33). Coaches noted that pre-session feedback was less common, but increasingly valued for adjusting training goals and managing expectations (10). Conversely, feedback during and after sessions was more frequently employed to support technical, physical, and psychological adaptation (6). These findings align with other studies (34, 35), which identified similar recovery practices, such as stretching, naps, and massage therapy, used during training and competition.

Strength and power capacities were also systematically developed throughout the season via dryland training, including maximum strength, core, power-endurance, and neuromuscular activation exercises. These methods, as reported by Gonzalez-Rave et al. (30), contributed to measurable improvements in lower-body power and overall swimming performance. Coaches' understanding of training quality reflected a multidimensional concept, including factors such as task planning, swimmer motivation, time efficiency, and technical-physical balance. Coaches define training quality as “*training that is effective, efficient, and aligned with short-, mid, and long-term goals*.” Most coaches favored in-person or hybrid supervision models, reinforcing the value of continuous coach-athlete interaction as a driver of adaptive and high-quality training (10).

Warm-up routines were consistently structured and integrated both dryland activation and in-water technical elements, including speed and neuromuscular preparation. These practices aligned with evidence supporting the use of combined activation protocols to enhance readiness and performance (36), emphasizing the benefit of combining dryland mobility and activation with technical swim work and speed activation. These findings are also supported by McGowan et al. (37), who reported that elite swimming coaches frequently implement integrated warm-up routines combining dryland and water-based activities, tailored to session goals and athlete feedback, to optimize readiness and performance.

Taken together, these results illustrate that planning in high-performance swimming is not only a matter of technical modeling but a complex negotiation between structure, context, and interpersonal dynamics. Coaching decisions are shaped by variables such as access to facilities, athlete availability, cultural expectations, and competition schedules. The coaches in our study appeared to apply periodization not simply as a model, but as a flexible tool to manage uncertainty and adapt to environmental constraints. This interpretation is consistent with prior work emphasizing the socially situated nature of coaching knowledge (26, 27). In this sense, our findings extend current literature by offering a detailed description of how Spanish national coaches adapt global periodization models to their socio-institutional realities—a perspective that remains underexplored in existing studies.

This study has several limitations that should be acknowledged. First, the relatively small and heterogeneous sample may limit the generalizability of findings. Nonetheless, the use of purposive sampling ensured that participants had substantial and relevant coaching experience. Second, while the questionnaire captured both practices and beliefs, it limited our ability to explore the personal meanings behind their coaching decisions in depth. Third, variability in participants' professional and academic backgrounds likely influenced how key concepts—such as periodization, training quality, and feedback—were interpreted. This diversity mirrors real-world variation but introduces potential inconsistency in responses.

Future studies could also benefit from mixed-method approaches combining questionnaires with semi-structured interviews to gain deeper insights into coaching beliefs. Investigating how planning practices relate to actual performance outcomes in competition could also provide valuable information. Finally, incorporating swimmers' perspectives, particularly on perceived load, feedback received, and planning transparency, would offer a more holistic understanding of the coach-athlete dynamic.

## 5 Practical applications

The findings of this study offer valuable insights into swimming coaches and other professionals involved in athletic preparation. Both traditional and block periodization models were equally common among coaches, suggesting that there is no single “best” model and that training programs should be adapted to individual swimmers' needs and competition schedules. Coaches should consider integrating phases that focus on technical skills and physiological adaptations, regardless of the chosen periodization model. Monitoring methods, such as encoders and subjective scales, are frequently used by coaches and can support more individualized training adjustments. Additionally, consistent and individualized feedback during and after training sessions can enhance the swimmer's motivation and technical improvements. These results encourage the adoption of flexible planning strategies that balance scientific principles with practical realities in the training environment.

## 6 Conclusions

National-level swimming coaches predominantly use traditional periodization models, structured into distinct phases (general, specific, and competitive), with a strong emphasis on individualization and performance optimization. Their methodological approach integrates both objective and subjective indicators to monitor and manage training, prioritizing quality through physiological, metabolic, technical, and cognitive adaptations.

Coaches also adopt an integrated training process that includes adjustments before, during, and after each session, with swimmer collaboration particularly evident in recovery strategies. Key elements such as warm-up routines, tapering, and targeted strength and speed work are strategically implemented to enhance the training outcomes. Based on the prevailing trends identified, we recommend the use of individualized tapering periods lasting 7 and 21 days, particularly for national and elite-level swimmers, to maximize peak performance during major competitions.

## Data Availability

The original contributions presented in the study are included in the article/Supplementary Material, further inquiries can be directed to the corresponding author.
