# Supplementary material for: Swimming coaches' perceptions and practices on periodization, performance monitoring, and training management
Source: Front Sports Act Living. 2025 Aug 22;7:1642020. doi: 10.3389/fspor.2025.1642020 (PMC12411453; doi:10.3389/fspor.2025.1642020)
Supplement: Supplementary file 1 [file Datasheet1.pdf]

ANALYSIS OF THE WORK PLANNING OF SWIMMING COACHES

This questionnaire addresses questions related to the quality of training, which is defined as the degree of excellence related to how the training process or training sessions are carried out to optimize adaptations and/or improve overall performance.

Our purpose is not to evaluate or give an opinion on your work, our only objective is to collect information on the training process of athletes oriented to swimming performance, in order to deepen the knowledge and improve the existing information on this topic of study. This questionnaire is part of a line of research on the factors that influence the swimming coach for the planning of the season. The information obtained will be communicated to the participating coaches, always globally and maintaining the anonymity of the data obtained.

Thank you in advance for your participation.

PART 1. Coach's personal details

This data will be completely anonymous.

1. In which country do you live and train?

____

2. Years of experience

____

3. Level of Education/Education (you can choose more than one option)

☐ Certified coach by your Swimming Federation.

☐ Bachelor's degree in Sports Science (or related).

☐ Bachelor of Education (or related).

☐ Graduated in another sector.

☐ Master's degree in Sports Science (or related).

☐ Master's degree in another sector.

☐ PhD in Sports Science (or related).

☐ PhD in another sector.

☐ Other:

4. Category you primarily train to (you can choose more than one option)
    - ☐ Alevín
    - ☐ Children
    - ☐ Junior
    - ☐ Absolute
5. Best time (in seconds) of your best athlete. Specify your gender and category

    ____

6. Best result (position) of their best athlete. Specify the championship.

    ____

7. What is the style in which your best athlete trains/competes?
    - ☐ Freestyle (50, 100, 200, 400, 800 and 1500 m)
    - ☐ Backstroke (50, 100 and 200 m)
    - ☐ Breaststroke (50, 100, 200 m)
    - ☐ Butterfly (50, 100, 200 m)
    - ☐ Single style (200, 400 m)
    - ☐ Freestyle relay (4x50, 4x100 and 4x200 m)
    - ☐ Style relay (4x50 and 4x100 m)

PART 2. Holistic Process Quality

Below you will find questions about periodization, scheduling, and phase adjustment of your swimmers for a full season.

1. We define training quality as the degree of excellence related to how the training process or training sessions are executed to optimize adaptations and/or overall performance.

    What do you mean by training quality?

    ____

    ____

____

2. What would be the characteristics for a good quality workout?

3. How do you plan the season?
   - ☐ Individually
   - ☐ With the swimmers (Co-designers)
4. Do you take into account the swimmer's environment when carrying out the programme? (e.g. work, study, free time, family, ...)
   - ☐ Yes
   - ☐ Yes
5. What kind of periodization do you typically use with your top swimmers?
   - ☐ Traditional periodization (high-volume training during the preparation period. As the season progresses and the competitive period is passed, volume decreases and the focus is mainly on intensity)
   - ☐ Block periodization (on one or more specific characteristics (i.e., concentrated load), such as strength training, which is followed by the use of the residual effect to enhance the subsequent training block, such as speed-strength)
6. How do you divide the season? (i.e., what you call each of the phases and how many weeks they last) (e.g. introductory, general, specific, competitive, transitory, accumulation, transformation, realization phase, ...)

7. How many days before the competition does the de-intensity phase begin?

8. Can you explain your reduction protocol for your de-escalation phase? (i.e. % of volume reduction, number of sessions, session intensity, ...)

9. What is the goal of "force" in its Preparatory/General/Accumulation phase?

   (From now on we will use the terms in the image to refer to these capabilities)

	Strength -Endurance	Strength	Strength - Speed	Speed - Strength
**Meaning**	Ability to produce force over an extended period under load.	Ability to exert force to overcome resistance.	Ability to produce maximum force in a short period of time.	The greatest amount of force developed in a short period of time.
**Work intensity**	Body weight (BW)	80-100% of 1RM<br>0.23 – 0.5 m/s	30-70% of 1RM<br>0.5 – 1 m/s	<30% of 1RM<br>1 – 1.5 m/s

10. What importance do you give within the Preparatory/General/Accumulation phase of your strength work to each of the following "strength capabilities"?

    (From 1 to 10) (Mark with an X)

	**1**	**2**	**3**	**4**	**5**	**6**	**7**	**8**	**9**	**10**

**Strength-Endurance**										
**Strength**										
**Strength-Speed**										
**Speed-Strength**										

11. What kind of resources does the development of these capacities use? (e.g. sledding, medicine ball, elastic bands, sandbag, hurdles, bodyweight work, barbell front squat, barbell press, ...)
12. ____

____

____

____

____

13. What is the objective of "force" in its Specific/Transformation phase? (From now on we will use the terms in the image to refer to these capabilities)

	Strength -Endurance	Strength	Strength - Speed	Speed - Strength
**Meaning**	Ability to produce force over an extended period under load.	Ability to exert force to overcome resistance.	Ability to produce maximum force in a short period of time.	The greatest amount of force developed in a short period of time.
**Work intensity**	Body weight (BW)	80-100% of 1RM<br>0.23 – 0.5 m/s	30-70% of 1RM<br>0.5 – 1 m/s	<30% of 1RM<br>1 – 1.5 m/s

____

____

____

____

____

____

14. What importance do you give within the Specific/Transformation phase of your strength work to each of the following "strength capabilities"? (From 1 to 10) (Mark with an X)

	**1**	**2**	**3**	**4**	**5**	**6**	**7**	**8**	**9**	**10**
**Strength-Endurance**										
**Strength**										
**Strength-Speed**										
**Speed-Strength**										

15. What kind of resources do you use to develop these skills? (e.g. sledding, medicine ball, elastic bands, sandbag, hurdles, bodyweight work, barbell front squat, barbell press, ...)

16. What is the objective of "strength" in its Competitive phase? (From now on we will use the terms in the image to refer to these capabilities)

	**Strength -Endurance**	**Strength**	**Strength - Speed**	**Speed - Strength**
**Meaning**	Ability to produce force over an extended period under load.	Ability to exert force to overcome resistance.	Ability to produce maximum force in a short period of time.	The greatest amount of force developed in a short period of time.
**Work intensity**	Body weight (BW)	80-100% of 1RM<br>0.23 – 0.5 m/s	30-70% of 1RM<br>0.5 – 1 m/s	<30% of 1RM<br>1 – 1.5 m/s

17. What importance do you give within the Competitive phase of your strength work to each of the following "strength capabilities"? (From 1 to 10) (Mark with an X)

	**1**	**2**	**3**	**4**	**5**	**6**	**7**	**8**	**9**	**10**
**Strength-Endurance**										
**Strength**										
**Strength-Speed**										
**Speed-Strength**										

18. What kind of resources do you use to develop these skills? (e.g. sledding, medicine ball, elastic bands, sandbag, hurdles, bodyweight work, barbell front squat, barbell press, ...)

19. What is the objective of swimming in its Preparatory/General/Accumulation phase? (From now on we will use the terms in the image to refer to these capabilities)

	Speed	Strength	Flexibility	Endurance	Coordination
**Meaning**	Ability to exert maximum speed of reaction and movement.	Ability to generate propulsion that moves the body forward.	Ability to perform movements with a wide range of motion and improve technique.	Ability to maintain an optimal swimming pace for longer and avoid fatigue.	Ability to coordinate or organize body movements to achieve maximum speed with the least resistance.

What importance do you give within the Preparatory/General/Accumulation phase of your swimming work to each of the following "swimming abilities"?

	**1**	**2**	**3**	**4**	**5**	**6**	**7**	**8**	**9**	**10**
**Speed**										
**Strength**										
**Flexibility**										
**Endurance**										
**Coordination**										

20. What kind of resources do you use to develop these skills? (e.g. weighted vests, pull buoy, foam rollers, elastic straps, weights, ...)

21. What is the goal of swimming in its Specific/Transformation phase? (From now on we will use the terms in the image to refer to these capabilities)

	Speed	Strength	Flexibility	Endurance	Coordination
Meaning	Ability to exert maximum speed of reaction and movement.	Ability to generate propulsion that moves the body forward.	Ability to perform movements with a wide range of motion and improve technique.	Ability to maintain an optimal swimming pace for longer and avoid fatigue.	Ability to coordinate or organize body movements to achieve maximum speed with the least resistance.

22. What importance do you give within the Specific/Transformation phase of your swimming work to each of the following "swimming abilities"?

	**1**	**2**	**3**	**4**	**5**	**6**	**7**	**8**	**9**	**10**
**Speed**										
**Strength**										
**Flexibility**										
**Endurance**										
**Coordination**										

23. What kind of resources do you use to develop these skills? (e.g. weighted vests, pull buoy, foam rollers, elastic straps, weights, ...)

24. What is the objective of swimming in its Competitive phase? (From now on we will use the terms in the image to refer to these capabilities)

	Speed	Strength	Flexibility	Endurance	Coordination
**Meaning**	Ability to exert maximum speed of reaction and movement.	Ability to generate propulsion that moves the body forward.	Ability to perform movements with a wide range of motion and improve technique.	Ability to maintain an optimal swimming pace for longer and avoid fatigue.	Ability to coordinate or organize body movements to achieve maximum speed with the least resistance.

25. What importance do you give within the Competitive phase of your swimming work to each of the following "swimming abilities"?

	**1**	**2**	**3**	**4**	**5**	**6**	**7**	**8**	**9**	**10**
**Speed**										
**Strength**										
**Flexibility**										

**Endurance**										
**Coordination**										

26. What kind of resources do you use to develop these skills? (e.g. weighted vests, pull buoy, foam rollers, elastic straps, weights, ...)

____

____

____

____

____

PART 3. Quality of the specific training session

Below you will find questions about managing your sessions.

1. How do you track and ensure the quality of your swimmers' training?
   - ☐ In person
   - ☐ Online
   - ☐ Hybrid (in-person and online)
2. Do you adjust the content of your session with pre-session measurements?
   - ☐ Yes
   - ☐ Yes
3. What kind of measurements do you use before your sessions to adjust the content of your sessions? (you can choose more than one option)
   - ☐ Physics (intervals, plyometrics, ...)
   - ☐ Techniques (technical exercises, ...)
   - ☐ Mental/Cognitive (sleep quality, ...)
   - ☐ None
   - ☐ Another: ____

4. What kind of measurements do you use to manage your sessions?

- ☐ Objective measurements (e.g. heart rate, duration, lactate...)
- ☐ Subjective measurements (e.g. RPE, questionnaires, ...)
- ☐ Both measurements (objective and subjective)

5. What kind of resources do you use to quantify and manage the load of your top swimmers during sessions? (e.g. heart rate, swimmer feedback, sleep, ...)

____

____

____

____

____

6. How do you organize the session with your swimmers?

- ☐ After the warm-up, the session is carried out without any prior explanation.
- ☐ After the warm-up, the objective of the session and the reason for choosing it are explained before starting the main part.
- ☐ The objective of the session is modified before starting it depending on the athlete's condition.
- ☐ Other: ____

7. Could you explain your warm-up protocol for your pool sessions? (e.g., 1. Muscle activation; 2. Mobility; 3. Progressive repetitions, ...)

____

____

____

____

____

8. How is your load management in the session? (you can choose more than one option)

- ☐ The load is adjusted based on subjective measurements (perceptions and feedback from swimmers)
- ☐ The load is adjusted based on objective measurements (heart rate, time, ...)
- ☐ Load does not change during the session (maintains the load set for the session)

9. Do you try to get your athletes to perform their training by focusing on the activity itself and its technical execution (technically correct regardless of the task)? How do you check it? (e.g. by observing their technique, asking the athletes, ...)

____

____

____

____

____

10. Do you control the speed/intensity of your swimmers during the session? (i.e. monitor the speed to verify that they are training at the correct intensity)

____

____

____

____

____

11. Do you use other elements besides the stopwatch and distance to verify that the athlete is doing the work at the right speed in the sessions? How? (e.g. Swim-Master...

____

____

____

____

____

12. When do you provide feedback to your athletes in the session? (you can choose more than one option)

- ☐ Feedback before the session
- ☐ In-session feedback
- ☐ Post-session feedback

13. What kind of feedback do you provide to your swimmers in the sessions? Why?

____

____

____

____

____

14. Do you consider the deviation between the objective of the session and its execution when analyzing the session? How? (e.g. more rest days, change of the goal of the next session, etc.)

____

____

____

____

____

15. How do you organise the recovery protocol in terms of active recoveries (high-intensity pace runs between days) and passive recoveries (massages, stretching, ...)? We assume that sleep and nutrition are used because they are critical to the recovery process (you can choose more than one option).

- ☐ They carry out a recovery protocol prescribed by the coach
- ☐ They carry out a recovery protocol prescribed by themselves
- ☐ They carry out a recovery protocol established between the athlete and the coach
- ☐ Another: ____

16. What kind of recovery protocol do you provide to your athletes? Why?

____

____

____

____

____

17. What test do you use to measure the strength and conditioning performance of your top swimmers on test days? (e.g. F-V profile, 1RM, plyo, CMJ, ...)

____

____

____

18. Which test do you use to measure the swimming performance of your top swimmers on test days? (e.g. incremental intensity tests (7 x 200m), ...)

The questionnaire has now ended. If you have any other comments or requests, please email Cristina.Cano7@alu.uclm.es and we will be in touch.

Thank you very much.

Supplementary Material 1. Questionnaire about analysis of the work planning of swimming coaches.
